# Trends and Outcomes of Readmissions Following Post-Procedural Stroke in Patients Undergoing Transcatheter Edge-to-Edge Repair: Insights From the National Readmission Database (2016-2020)

**DOI:** 10.7759/cureus.78512

**Published:** 2025-02-04

**Authors:** Nirav Patel, Yasar Sattar, Abdul Rasheed Bahar, Daniya Muhammad Haroon, Krutarth Pandya, Shafaqat Ali, Sadaf Fakhra, Neel N. Patel, M. Chadi Alraies

**Affiliations:** 1 Internal Medicine, Saint Michael's Medical Center, Newark, USA; 2 Internal Medicine, Icahn School of Medicine at Mount Sinai, New York, USA; 3 Internal Medicine, Wayne State University, Detroit, USA; 4 Internal Medicine, Bahria University Medical and Dental College, Karachi, PAK; 5 Internal Medicine, Cleveland Clinic, Cleveland, USA; 6 Internal Medicine, Louisiana State University Health Sciences Center, Shreveport, USA; 7 Internal Medicine, University of Nevada Las Vegas School of Medicine, Las Vegas, USA; 8 Internal Medicine, New York Medical College/Landmark Medical Center, Woonsocket, USA; 9 Cardiology, Wayne State University Detroit Medical Center, Detroit, USA

**Keywords:** nrd, outcomes, post-procedure, stroke, teer

## Abstract

Introduction

Transcatheter edge-to-edge repair (TEER) procedure for the repair of significant symptomatic mitral regurgitation has become increasingly popular in recent years. Stroke is a well-known complication of many surgical procedures. However, the association of stroke on outcomes of TEER in patients has not been adequately reported in the literature.

Methods

We queried the National Readmission Database from 2016 to 2020 using ICD-10 codes to identify the patients admitted for TEER. The patients were divided into two groups: patients with stroke and patients without stroke. Outcomes were assessed between two cohorts at index admissions and readmissions due to stroke.

Results

A total of 16,719 patients were admitted for TEER procedure, and 97 patients were diagnosed with new onset acute stroke/cerebrovascular accident (CVA). The most common comorbidities in the study population admitted with acute CVA were hypertension, hyperlipidemia, history of nicotine use, and coronary artery disease. On multivariate regression analysis, patients admitted with acute CVA compared to the patients without acute CVA had significantly higher odds of in-hospital mortality, acute kidney injury, post-procedural bleeding, acute myocardial infarction, and mechanical circulatory support.

Conclusion

Patients in the acute CVA group had higher rates of readmissions, mean length of stay in the hospital, and higher healthcare burden.

## Introduction

Transcatheter edge-to-edge repair (TEER) using MitraClip™ implantation is a percutaneous technique that achieves mitral valve repair by approximating the mitral leaflets, effectively reducing significant symptomatic mitral regurgitation in selected patients [1]. The procedure is performed with a catheter inserted through the femoral vein, reaching the left side of the heart, and attaching the clip to the leaking portions of the mitral valve [2]. TEER is a widely used percutaneous approach worldwide [3,4] and has been applied clinically since 2003 [5]. Although surgery is effective, it is associated with significant morbidity and mortality, especially in the presence of advanced age or significant comorbidity. TEER is an effective treatment option for patients with significant mitral regurgitation who are considered high-risk for surgery, as it offers a lower risk of thromboembolism and infection, demonstrates favorable durability, and may improve survival in patients who are unsuitable for surgical intervention [6]. Stroke is a well-known complication of many surgical procedures. However, the association of stroke with outcomes of TEER procedure in patients has not been adequately reported in the literature. We took detailed consideration of all the comorbidities in the patients. We conducted a study to depict the impact of stroke on readmissions in 30 days, 90 days, and 180 days, as well as outcomes after TEER.

## Materials and methods

Study design and population

This is a retrospective cohort study derived from the National Readmission Database (NRD) sponsored by the Agency for Healthcare Research and Quality (AHRQ) as a part of the Healthcare Cost and Utilization Project (HCUP). The NRD provides national information on hospital readmissions and is the largest all-payer and uninsured patient readmissions database in the United States. We queried the NRD (2016 to 2020) and identified patients admitted for TEER using the International Classification Disease, 10th Revision, Clinical Modification (ICD-10 CM) (Appendices A-D). Patients were categorized into two sub-groups: those with stroke and those without stroke. Stroke was defined as an event occurring within 30 days following the TEER procedure.

Baseline characteristics

Comprehensive data on patients' medical history were collected, including cerebrovascular events (stroke), coagulopathy, congestive heart failure (CHF), prior myocardial infarction (MI), peripheral vascular disease (PVD), valvular heart disease, liver disease, lymphoma, electrolyte imbalances, metastatic cancer, paralysis, psychiatric disorders, pulmonary circulatory disorders, non-metastatic solid tumors, obesity, unintentional weight loss, and substance use such as smoking, alcohol, and drug consumption. Additionally, chronic comorbid conditions, including diabetes (with and without complications), hypertension, hyperlipidemia, hypothyroidism, chronic kidney disease, chronic pulmonary disease, and depression, were evaluated. Previous cardiovascular procedures, such as percutaneous coronary intervention (PCI) and coronary artery bypass grafting (CABG), were also considered in the analysis.

Study outcomes

The objective of this study is to evaluate the impact of post-procedural stroke on outcomes and readmissions in patients who underwent the TEER procedure. Patients with a prior history of cerebrovascular accidents (CVAs) were identified based on documented medical records, and a history of stroke was defined as any prior ischemic or hemorrhagic event before TEER. Additionally, stroke events were categorized based on established classifications, including transient ischemic attack (TIA), major or minor stroke, and functional outcomes such as disability status. The major outcomes assessed during index hospitalization were in-hospital mortality, acute kidney injury (AKI), heart failure, stroke, MI, the requirement for mechanical circulatory support (MCS), and major adverse cardiac events (MACEs).

Statistical analysis

Pearson’s chi-square test was used for categorical variables, and a t-test was used for continuous variables. A p-value of <0.05 was considered statistically significant. Cardiovascular outcomes were assessed between both cohorts at index admission and readmissions at 30, 90, and 180 days. Stata v.17 (StataCorp, College Station, TX) was used for analysis.

## Results

Baseline demographics and comorbidities

**Figure 1:**
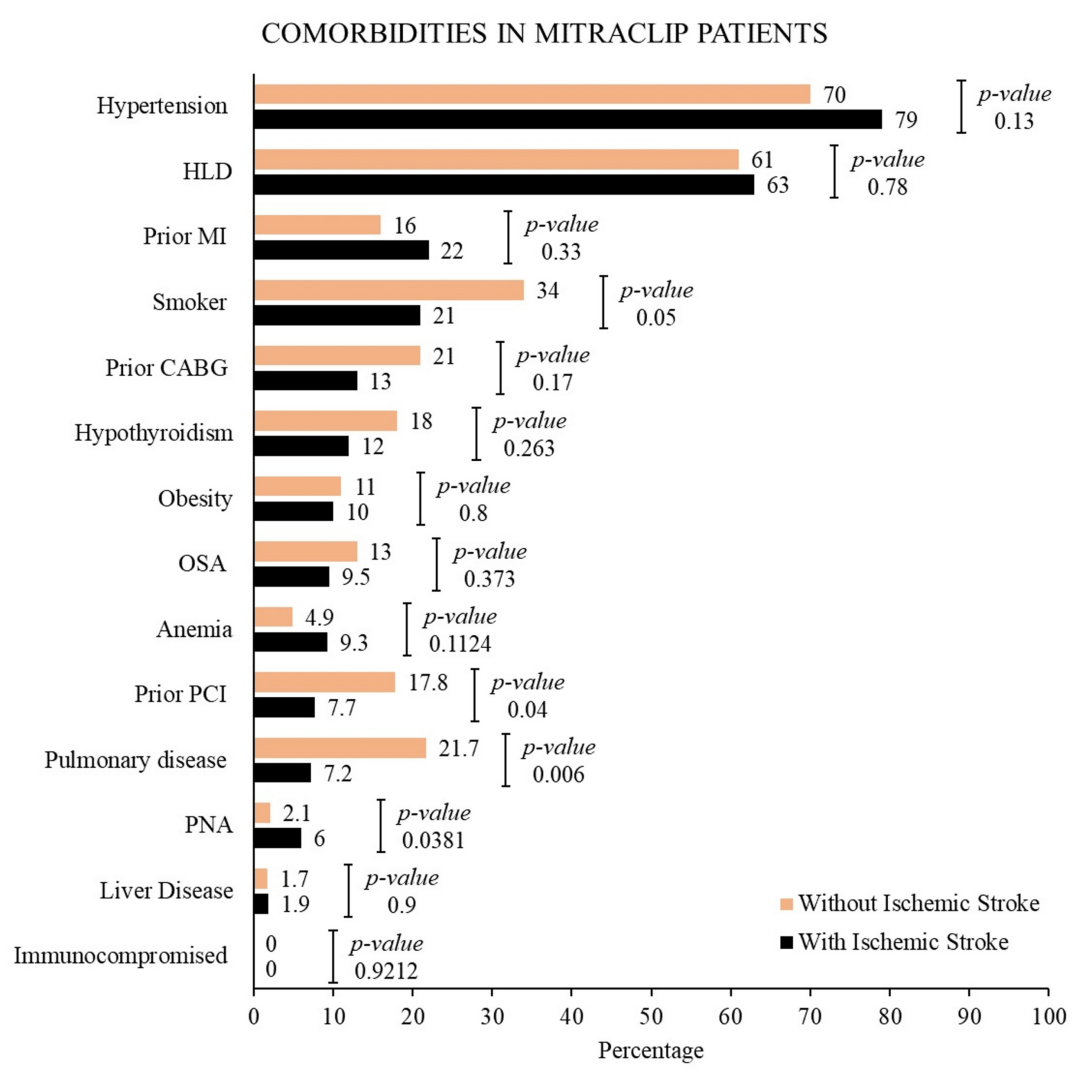
Baseline comorbidities in MitraClip patients HLD, hyperlipidemia; MI, myocardial infarction; CABG, coronary artery bypass grafting; OSA, obstructive sleep apnea; PCI, percutaneous coronary intervention; PNA, pneumonia

We identified a total of 28,997 patients who underwent TEER with MitraClip™ between January 11, 2016, and December 31, 2020, from an unweighted sample using the NRD. Of these, 97 (0.58%) patients were diagnosed with new onset acute stroke/CVA. We compared our study’s primary and secondary outcomes among TEER patients admitted with and without acute CVA. Patients with acute CVA were relatively younger (mean age 76 years vs. 77 years) and had relatively higher weekend admissions (10% vs. 4%) and same-day transfers (14.5% vs. 2.8%). In addition, hypertension (79%), hyperlipidemia (63%), history of nicotine use (21%), and history of coronary artery disease (CAD) (22%) were the most common comorbidities in the study population admitted with acute CVA. Baseline demographics and comorbidities are described in Tables 1, 2 and Figure 1.

**Table 1:** Baseline characteristics for the study groups

	TEER without stroke, N (%)	TEER with stroke, N (%)	p-Value	Chi-square value	F-value
Mean age ± SD	77.6 ± 10	76.7 ± 12	-	-	-
Female	13,232 (45)	57 (58)	0.08	5.16	2.94
Rehab transfer			0.25	2.12	1.35
No rehab transfer	28,735 (99)	95 (98)			
Rehab transfer	165 (0.6)	2 (2)			
State resident status			0.96	0.005	0.001
Non-resident	2,786 (10)	10 (10.3)			
Resident	26,113 (90)	87 (89.7)			
Same day event			<0.000	60.78	11.38
Not a transfer or same-day event	27,449 (95)	76 (78)			
Same-day 1 discharge involved	823 (2.8)	14 (14.5)			
Same-day 2 discharges involved	306 (1)	1 (0.01)			
Same-day 3 discharges involved	147 (0.5)	3 (0.3)			
Same-day 4 discharges involved	174 (0.6)	3 (3)			
Median household income			0.7279	2.31	0.42
0-25th percentile	6,309 (22)	26 (26)			
26-50th percentile	7,343 (26)	20 (20)			
51-75th percentile	7,775 (27)	29 (30)			
76-100th percentile	7,147 (25)	21 (21)			
Hospital bed size			0.4753	5.11	0.64
Small	1,176 (4)	0 (0)			
Medium	5,939 (21)	16 (16)			
Large	21,784 (75)	82 (84)			
Control/ownership of the hospital			<0.000	13.32	4.20
Government, nonfederal	2,365 (80)	18 (19)			
Private, non-profit	23,119 (80)	73 (75)			
Private, invest own	3,416 (12)	6 (6)			
Urban-rural hospital designation			0.7288	3.71	0.21
Large metropolitan	20,022 (69)	59 (60)			
Small metropolitan	8,737 (30)	39 (39)			
Micropolitan	137 (0.4)	0 (0)			
Teaching status of urban hospitals			0.8198	1.12	0.08
Metropolitan non-teaching	2,733 (9.5)	6 (6.6)			
Metropolitan teaching	26,027 (90)	91 (93)			
Metropolitan teaching	139 (0.5)	0 (0)			
Admission day			<0.000	8.25	2.77
Monday-Friday	27,761 (96)	88 (90)			
Saturday-Sunday	1,139 (4)	9 (10)			
Primary expected healthcare cost payer			0.6516	6.94	0.65
Medicare	25,221 (87)	79 (81)			
Medicaid	700 (2.4)	4 (4.5)			
Private	2,456 (8.5)	14 (14.5)			
Self-pay	126 (0.4)	0 (0)			
No charge	16 (0.05)	0 (0)			
Other	369 (1.2)	0 (0)			

**Table 2:** Baseline comorbidities for the study groups MI, myocardial infarction; PCI, percutaneous coronary interventions; CABG, coronary artery bypass graft; OSA, obstructive sleep apnea

Comorbidities	TEER without stroke, N (%)	TEER with stroke, N (%)	p-Value		Chi-square value	F- value
Smoker	9,835 (34)	20 (21)		0.05	6.37	3.82
Prior MI	4,733 (16)	21 (22)		0.33	1.76	0.93
Prior PCI	5,153 (17.8)	7 (7.7)		0.04	5.85	4.12
Prior CABG	6,092 (21)	12 (13)		0.17	3.53	1.91
OSA	3,791 (13)	9 (9.5)		0.373	0.98	0.79
Pulmonary disease	6,283 (22)	7 (7.2)		0.006	10.0	7.5
Immunocompromised	5 (0.0)	0 (0)		0.9212	0.01	0.01
Hypothyroidism	5,088 (18)	12 (12)		0.263	1.7	1.25
Anemia	1,426 (4.9)	9 (9.3)		0.1124	3.5	2.52
Pneumonia	628 (22)	6 (6)		0.0381	5.7	4.30
Liver disease	488 (1.7)	2 (1.9)		0.9	0.02	0.01
Hypertension	20,247 (70)	77 (79)		0.13	3.25	2.25
Hyperlipidemia	17,582 (61)	61 (63)		0.78	0.14	0.07
Obesity	3,218 (11)	10 (10)		0.8	0.09	0.05

 In-hospital complications

Complications for TEER patients with and without acute CVA are outlined in Tables 3, 4.

**Table 3:** Outcomes in TEER patients with versus without stroke (unmatched) TEER, transcatheter edge-to-edge repair; MCS, mechanical circulatory support; MACCE, major adverse cardiac and cerebrovascular events

Outcomes	TEER without stroke (%)	TEER with stroke (%)	p-Value
Died during hospitalization	2.1	23.1	<0.001
Acute kidney injury	15.1	51.2	<0.001
Heart failure	83.3	85.8	0.656
Myocardial infarction	1.7	14.7	<0.001
MCS	2.3	11.9	<0.001
MACCE	6.7	100	<0.001
Post-procedural bleeding	1.6	5.1	0.015
Cardiac tamponade	0.5	2.6	0.007

**Table 4:** Outcomes in TEER patients with versus without stroke (propensity-matched outcomes) TEER, transcatheter edge-to-edge repair; SCA, sudden cardiac arrest; MCS, mechanical circulatory support; MACE, major adverse cardiac events

Outcomes	TEER without stroke (%)	TEER with stroke (%)	p-Value
In-hospital mortality	1.7	22.4	0.001
Acute kidney injury	19	52	<0.000
Heart failure	79	86	0.33
Myocardial infarction	0	13.8	0.003
Post-procedure bleeding	0	6.9	0.042
SCA	5.2	27.6	0.001
MCS	0	10.3	0.012
MACE	5.2	100	<0.000
Cardiac tamponade	0	3.5	0.15
Cardiogenic shock	3.5	24	0.001

As compared to patients without acute CVA, TEER patients admitted with acute CVA had higher inpatient mortality (23.1% vs. 2.1 % p=0.000), had AKI (51.2% vs. 15.1% p=0.000), had acute MI (14.7% vs. 1.7% p=0.000), required more MCS (11.9% vs. 2.3% p=0.000), had post-procedural bleeding (5.1% vs. 1.6% p=0.015), and developed more cardiac tamponade (2.6% vs. 0.5% p=0.007). In contrast, there was no significant difference in CHF between two patient populations.

Crude outcomes on univariate regression analysis

**Figure 2:**
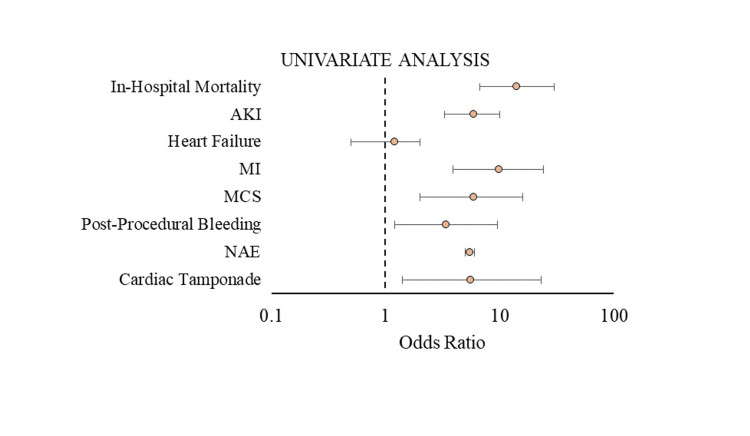
Univariate Cox regression analysis of the clinical outcome in patients with stroke who underwent TEER procedure AKI, acute kidney injury; MI, myocardial infarction; MCS, mechanical circulatory support; NAE, net adverse events; TEER, transcatheter edge-to-edge repair

TEER patients admitted with acute CVA compared to the patients without acute CVA had significantly higher odds of unadjusted in-hospital mortality (unadjusted OR [uOR] 14, 95% CI 6.7- 30, p<0.001), AKI (uOR 5.89, 95% CI 3.3-10, p<0.001), acute MI (uOR 9.8, 95% CI 3.9-24, p<0.001), post-procedural bleeding (uOR 3.4, 95% CI 1.2-9.5, p=0.002), sudden cardiac arrest (uOR 8.1, 95% CI 4.3-15, p<0.001), and cardiac tamponade (uOR 5.6, 95% CI 1.4-23, p=0.02) (Figure 2).

In contrast, the two groups had no statistically significant difference regarding MCS and CHF (Table 5).

**Table 5:** Comparison of outcomes in TEER patients with versus without stroke (univariate regression analysis) OR, odds ratio, LCI, lower confidence interval; UCI, upper confidence interval; SCA, sudden cardiac arrest; MCS, mechanical circulatory support; MACE, major adverse cardiac events; TEER, transcatheter edge-to-edge repair

Outcomes of univariate regression analysis	OR	LCI	UCI	p-Value
In-hospital mortality	14	6.7	30	<0.001
Acute kidney injury	5.89	3.3	10	<0.001
Heart failure	1.2	0.5	3	0.657
Myocardial infarction	9.8	3.9	24	<0.001
Post-procedure bleeding	3.4	1.2	9.5	0.02
SCA	8.1	4.3	15	<0.001
MCS	5.9	2	16	0.087
MACE	1	0.07	0.08	0
Cardiac tamponade	5.6	1.4	23	0.02

Multivariate regression analysis and propensity-matched cohort

**Figure 3:**
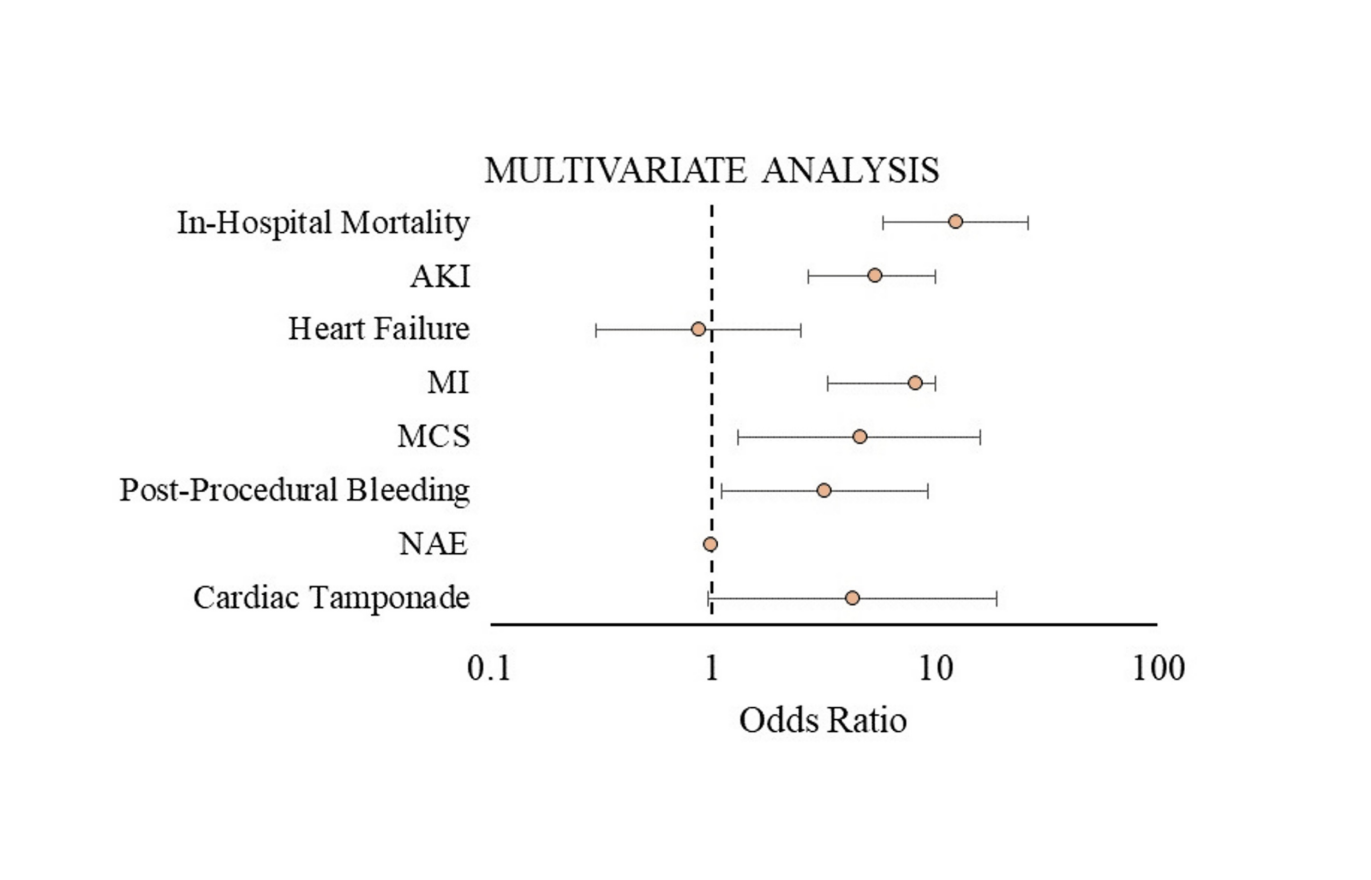
Multivariate Cox regression analysis of the clinical outcome in patients with stroke who underwent TEER procedure AKI, acute kidney injury; MI, myocardial infarction; MCS, mechanical circulatory support; NAE, net adverse events; TEER, transcatheter edge-to-edge repair

On multivariate regression analysis, TEER patients admitted with acute CVA compared to the patients without acute CVA had significantly higher odds of in-hospital mortality (OR 12.5, 95% CI 5.8-26, p<0.001), AKI (OR 5.4, 95% CI 2.7-10, p<0.001), post-procedural bleeding (OR 3.2, 95% CI 1.1-9.3, p=0.03), acute MI (OR 8.2, 95% CI 3.3 -19.9, p<0.001), and MCS (OR 4.6, 95% CI 1.3-16, p=0.002) (Figure 3).

There were no statistically significant differences between the two groups regarding cardiac tamponade and CHF (Table 6).

**Table 6:** Comparison of outcomes in TEER patients with versus without stroke (multivariate regression analysis) OR, odds ratio; LCI, lower confidence interval; UCI, upper confidence interval; MCS, mechanical circulatory support; TEER, transcatheter edge-to-edge repair

Outcomes of multivariate regression analysis	OR	LCI	UCI	p-Value
In-hospital mortality	12.5	5.8	26	<0.001
Acute kidney injury	5.4	2.7	10	<0.001
Heart failure	0.87	0.3	2.5	0.8
Post-procedure bleeding	3.2	1.1	9.3	0.03
Myocardial infarction	8.2	3.3	19.9	<0.001
MCS	4.6	1.3	16	0.02
Cardiac tamponade	4.3	0.96	19	0.057

**Figure 4:**
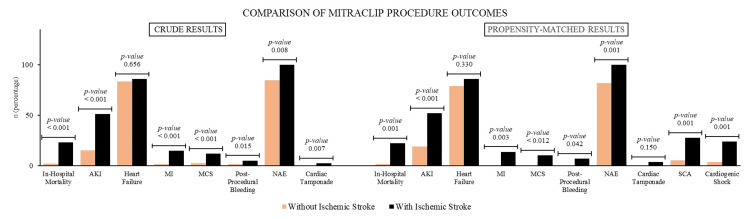
Comparison of MitraClip TEER procedure outcome in patients with and without ischemic stroke AKI, acute kidney injury; MI, myocardial infarction; MCS, mechanical circulatory support; NAE, net adverse events; SCA, sudden cardiac arrest; TEER, transcatheter edge-to-edge repair

Similarly, after propensity matching, the TEER group with acute CVA had higher AKI (52% vs. 19%, P = 0.000). After propensity matching, outcomes with frequencies <10 and percentages <1% were excluded from the final analysis (Figure 4).

## Discussion

Over the last two decades, there has been significant progress with innovations in structural cardiac interventions. The tremendous success of transcatheter aortic valve replacement (TAVR) was followed by TEER with MitraClip™ implantation, which was first approved in 2013 by the FDA with a new indication approved in 2019 [7,8].

TEER can increase the risk of stroke due to multiple factors. Potential factors include the transseptal puncture (TSP) technique, periprocedural emboli from acute thrombus, and hydrophilic polymer coating of the device, air, valve, or atrial tissue [9]. The TSP technique was developed by Ross, Braunwald, and Marrow in 1959 to allow direct measurement of left atrial pressure [10]. It has been widely used now for many structural cardiac interventions [11]. TSP typically heals within six months [12]. Patients can be at increased risk for the time being for stroke because of interatrial shunt. In the last three decades, the use of lubricious (hydrophilic and/or hydrophobic) polymer-coated devices has increased significantly by interventional physicians and vascular surgeons to access and treat a wider range of pathologies procedures, with TEER being one of them [13]. One of the drawbacks of these polymer coatings is the separation of the polymer coat during an interventional procedure. The hydrophilic polymer coating can adsorb water and become less slippery in an aqueous environment, leading to an increased risk of emboli [13]. Many studies have shown the risk of embolization from endovascular procedures including TAVR and strategies to reduce the same [14-19]. Approaches and techniques have been developed to decrease the risk of stroke after TEER. A cerebral protection device (CPD) is a device used during endovascular procedures to reduce the risk of embolization during these procedures. It has been used for structural and endovascular procedures such as TAVR and ventricular tachycardia ablations [20-23].

In a study on the histology of debris captured by CPDs during transcatheter valve-in-valve (VIV) implantation, transcatheter VIV was associated with the release of debris, and most debris comprised atrial and ventricular passage of transcatheter heart valve [24]. Catheter-based mitral valve interventions i.e., TEER, is a similar procedure where a device (clip) is used instead of a new valve; hence, this risk continues to be a threat if proper CPD is not used, which has been described in studies as well [25-27]. Initial studies and case reports have also shown the benefits of using CPDs such as Sentinel in mitral valve intervention [26,28-30].

TEER has achieved significant success in structural valvular interventions following the advancements made in TAVR, with the knowledge gained from TAVR playing a crucial role in its development and refinement. The increased risk of stroke associated with TAVR has been extensively studied, highlighting the role of hydrophilic coatings on endovascular devices, valvular manipulation, and other multifactorial mechanisms [31]. Several studies describe the role of different CPDs during TAVR procedures in clinical practice in preventing strokes [20,22,23,32]. However, their implementation in TEER remains limited, with a notable lack of supporting data.

Risk stratification

Independent factors affecting poor outcomes have been studied, and scores such as MitraScore have been developed and validated for risk stratification for patients undergoing TEER [33]. This score does not reflect the direct association with stroke though. Though the CHA2DS2-VASc score has been shown to be predictive for TAVR and TEER, the accuracy of this has been questioned [33,34]. Zweck et al. developed the MITRALITY score using machine learning to increase the accuracy of overall risk stratification [35]. Further real-world data from the implementation of these scores will help understand the reproducibility and reliability of these scores for risk stratification.

Implementation of our data

In our study, we tried to highlight the adverse clinical outcomes including significantly higher in-hospital mortality, the prevalence of AKI, MI, post-procedural bleeding, cardiac tamponade, and MCS requirements, as well as increased readmissions, length of stay (LOS), and healthcare burden among the patients who developed stroke after TEER compared to those who did not develop stroke. These comorbidities and factors should be taken into consideration while selecting patients for TEER who already have a higher risk of developing stroke otherwise. Using these data, patient risk stratification should be optimized by incorporating specific clinical parameters before proceeding with TEER to reduce the incidence of post-procedural stroke. Key parameters to consider include left ventricular ejection fraction, mitral regurgitation severity, renal function, atrial fibrillation status, and prior cerebrovascular events. Establishing appropriate cutoff values for these parameters can aid in better patient selection and procedural planning. Our study highlights the value of strategies for periprocedural risk reduction of stroke in terms of patient outcomes, as well as cost-effectiveness.

Limitations

This study is subject to several limitations inherent to the use of the NRD, including the reliance on administrative coding, which may introduce misclassification or underreporting of conditions. The NRD can give information about the index for TEER, but it does not provide information on how many total TEER procedures they underwent during the same or different hospitalization. The NRD also does not provide any information on periprocedural complications or if any measures of stroke prevention were used. From the NRD, we get information about whether the patients had concomitant comorbidities, but it cannot be said how controlled it was in terms of medical management. Patient compliance can be a major contributing factor as well, which was not known in our study. Because the information for medication was not available, it was not possible to comment on how many of the patients were on any anticoagulation or antiplatelets. Finally, as a retrospective observational study, causal relationships cannot be established, and residual confounding may persist despite statistical adjustments.

## Conclusions

Patients with acute CVA undergoing TEER had significantly worse in-hospital outcomes, including higher mortality and complication rates such as AKI, MI, bleeding, and MCS use. Stroke after TEER can be detrimental in terms of overall outcomes, quality of life, readmissions, LOS, and healthcare burden. Our study and data will help stratify the risk of stroke and create new scores to predict the risk of stroke among patients undergoing TEER. It will also help decide on aggressive strategies used peri-procedurally while keeping cost-effectiveness into consideration as well. Further clinical trials are needed to better understand the risk factors associated with the development of stroke among patients undergoing TEER.

## Appendices

Appendix A

**Table 7:** ICD-10 codes for cohort identification and stratification with major baseline comorbidities We used smart coding where software matches the first three main digits of ICD-10-CM coding SCA, sudden cardiac arrest; PAD, peripheral artery disease; OSA, obstructive sleep apnea; MI, myocardial infarction; CABG, coronary artery bypass grafting; PCI, percutaneous coronary intervention

Variable	ICD-10 CM Codes
Comorbidities analyzed	
Hyperlipidemia	D68.9,
Hypertension	I10, I1150, I1151, I1152, I1158, I1159
SCA	I46, I97, O29,004,007
Anemia	
Obesity	R34, R94, I63, I67, I69, N14, N17, N1, N99
Major bleeding	D62,I60,I61,62,R31,R04,D500,H313,H356,H431,H450,I312,I850,K250,K252,K254,K256,K260,K262,K264,K266,K270,K272,K274,K276,K280,K282,K284,K286,K625,K661,K920,K921,K922,S064,S065,S066,J942,K228F,K298A,K638B,K638C,K838F,K868G,I864A,H052A,S368D,G951A
PAD	I730, I7300, I7301, I731, I738, I7389, I739
OSA	G4733
Prior MI	I252
Prior CABG	Z951
Prior PCI	Z986, Z9861, Z9862
Liver disease	K700, K70,1 K7010, K7011, K702, K703, K7030, K7031, K704, K7040, K7041, K709, Q898, Q446, I820, R160, K7460, K710, K711, K7110, K7111, K712, K71,3 K714, K715, K7150, K7151, K716, K717, K718, K719, K760, K761, K762, K763, K764, K765, K766, K767, K768, K7681, K7689, K769
Illicit drugs	F191, F1910, F1911, F1912, F19120, F19121, F19122, F19129, F1914, F1915, F19150, F19151, F19159, F1916, F1917, F1918, F19180, F19181, F19182, F19188, F1919, F192, F1920, F1921, F1922, F19220, F19221, F19222, F19229, F19230, F19231, F19232, F19239, F1924, F1925, F19250, F19251, F19259, F1926, F1927, F1928, F19280, F19281, F19282, F19288, F1929, F199, F1990, F1992, F19920, F19921, F1992,2 F19929, F1993, F19930, F19931, F19932, F19939, F1994, F1995, F19950, F19951, F19959, F1996, F1997, F1998, F19980, F19981, F19982, F19988, F1999
Alcohol	F101, F1010, F1011, F1012, F10120, F10121, F10129, F1014, F1015, F10150, F10151, F10159, F1018, F10180, F10181, F10182, F10188, F1019, F102, F1020, F1021, F1022, F10220, F10221, F10229, F1023, F10230, F10231, F10232, F10239, F1024, F1025, F10250, F10251, F10259, F1026, F1027, F1028, F10280, F10281, F10282, F10288, F1029, F10920, F10921, F10929, F1094, F1095, F10950, F10951, F10959, F1096, F1097, F1098, F10980, F10981, F10982, F10988, F1099
Smoking	F17, F172, F1720, F17200, F17201, F17203, F17208, F17209, F1721, F17210, F17211, F17213, F17218, F17219, F1722, F17220, F17221, F17223, F17228, F17229, F1729, F17290, F17291, F17293, F17298, F17299, Z87891
Pneumonia	J180, J181, J182, J188, J189
Pulmonary disease	J98.4
Lower GI bleeding	K51011, K51211, K51311, K51411, K51511, K51811, K51911, K50011, K50111, K50811, K50911, Q2733, K922, K5701, K5711, K5713, K5731, K5733, K5741, J4542, K5751, K5753, K5781, K5791, K5793, K649, K922, K625, K5521, K6381, K55051, K55049, K55042, K55031, K55032, K55033, K55041, K55052, K55059, K55061, K55062, K55069, K520, K529, K640, K641, K642, K643, K645, K648, K649, C189, C218, C20, C785, C19, C179
Upper GI bleeding	K250, K251, K252, K254, K255, K256, K9281, K260, K261, K262, K264, K266, K31811, K270, K271, K272, K274, K275, K276, K280, K281, K282, K284, K285, K286, K2211, K226, K223, K2901, K2921, K2931, K2941, K2951, K2961, K2971, K2981, K2991, K920, K921, K9421, K9431, C159, C170, d130, d131, i8501, i8511, K5000, K50011, K50012, K50013, K50014, K50018, K50019, K5010, K50111, K50112, K50113, K50114, K50118, K50119, K5080, K5081, K50812, K50813, K50814, K50818, K50819, K5090, K50911, K50912, K509123, K50914, K50918, K50919
GI hemorrhage	K922
History of stroke	Z86.73

Appendix B 

**Table 8:** Outcomes analyzed and their specific ICD-10 codes used to identify them in the National Readmission Database

Outcomes Analyzed		
Variable Name	Variable Description	Corresponding ICD-10 Codes
Acute kidney injury	Acute kidney injury incidence during hospital course	R34, R94, I63, I67, I69, N14, N17, N1, N99
HFrEF	Presence of heart failure with reduced ejection fraction during hospitalization course	I502, I5021, I5022, I5020, I5023
HFpEF	Presence of heart failure with preserved ejection fraction during hospitalization course	I503, I5031, I5032, I5030, I5033
Stroke + TIA	Stroke or transient ischemic attack occurring during the entirety of the hospitalization course.	G46, G45, I63
Post-procedural stroke	Stroke event occurring after the procedure in hospitalization course	I9782, I97820, I97821
Myocardial infarction	The event occurred during hospitalization	I121, I121.0, I21.1, I21.2, I21.3, I21.4, I21.5, I21.6, I21.7, I21.8, I21.9, I22, I22.1, I22.2, I22.3, I22.4., I22.5, I22.6, I22.7, I22.8, I22.9
Mechanical circulatory support	Patients were put on mechanical circulatory support during hospitalization	5A02110, 5A02210, 5A0211D, 02HA3RZ, 5A02116, 5A0221D, 5A1522F, 5A1522G, 5A1522H, 5A15A2F, 5A15A2G, 5A15A2H, 5A15223
Major adverse cardiac and cerebrovascular events	Major adverse cardiovascular and cerebrovascular events (MACCE) include in-hospital mortality, myocardial infarction, cardiogenic shock, and stroke	R570, T8111XA, T8111XS, I46, O29, 004, 007, R34, R94, 63, I67, I69, N14, N17, N19, N99, I2101, I2102, I2109, I2111, I2119, I2121, I2129, I213, I214, R570, T8111XA, T8111XS, I46, I97, O29, 004, 007
TEER	Transcatheter edge-to-edge repair	02UG3JZ

Appendix C 

**Table 9:** ICD-10 CM codes for cohort identification and stratification with major baseline comorbidities We used smart coding where software matches the first three main digits of ICD-10-CM coding PFO, patent foramen ovale; HD, hemodialysis; HLD, hyperlipidemia; HTN, hypertension; MI, myocardial infarction; PCI, percutaneous coronary intervention; CABG, coronary artery bypass grafting; OSA, obstructive sleep apnea

Variable	ICD-10 CM Codes
PFO cohort	“Q211"
PFO occluder device	02U53JZ
HD	5A1D70Z, 5A1D80Z, 5A1D90Z
HTN	I10, I1150, I1151, I1152, I1158, I1159
Obesity	"E6601" "E6609" “E661" "E662" "E668" "E669" "Z6830" "Z6831" "Z6832" "Z6833" "Z6834" "Z6835" "Z6836" "Z6837" "Z6838" "Z6839" "Z6841" "Z6842" "Z6843" "Z6844" "Z6845" "E66" "Z684" "E6601" "E6609" "E661" "E662" "E668" "E669" "Z683" "Z684" "R939" "Z6854" "O99210" "O99211" "O99212" "O99213" "O99214" "O99215"
Smoking	F17, F172, F1720, F17200, F17201, F17203, F17208, F17209, F1721, F17210, F17211, F17213, F17218, F17219, F1722, F17220, F17221, F17223, F17228, F17229, F1729, F17290, F17291, F17293, F17298, F17299, Z87891
Prior MI	I252
Prior PCI	Z986, Z9861, Z9862
Prior CABG	Z951
OSA	G4733
Pulmonary disease	"J449" "J444" "J440" "J441"
Immunocompromised	“D840" "D841" "D848" “D849"
Hypothyroid	"E030" "E031" "E032" "E033" "E034" "E035" E038" "E039"
Anemia	"D501" "D508" "D509" "D500" "D510" "D511" "D512" "D513" "D518" "D519" "D520" "D528" "D529" "D53" "D530" "D531" "D532" "D538" "D539"
Pneumonia	J180, J181, J182, J188, J189
Liver disease	K700, K70,1 K7010, K7011, K702, K703, K7030, K7031, K704, K7040, K7041, K709, Q898, Q446, I820, R160, K7460, K710, K711, K7110, K7111, K712, K71,3 K714, K715, K7150, K7151, K716, K717, K718, K719, K760, K761, K762, K763, K764, K765, K766, K767, K768, K7681, K7689, K769

Appendix D 

**Table 10:** Common variable definitions of cardiovascular outcomes **In-built codes in the National Inpatient Sample database for outcomes AKI, acute kidney injury

Outcomes	Definition	ICD-10 Codes
Survived**	Male and female patients after PFO occluder device placement that survived during the index hospitalization	Not applicable
Died**	In-hospital mortality of Male and female patients after PFO Occluder device placement	Not applicable
AKI	Acute kidney injury after PFO occluder device placement	“N171”,”N172”,”N178”, ”N179”, ”N19”, “N170”
Stroke	Any stroke occurring during the admission after PFO occluder device placement	“I639”, “I638”, “I6359”, “I63549”, “I63219”, “I63212”, “I63211”, “I6320”, “I6309”, “I63039”, “I63032”, “I63031”, “I6302”, “I63019”, “I63012”, “I63011”, “I6300”
Post-procedural bleeding	Any major bleeding after PFO occluder device placement	"I97418" "I9742" "I97618" "I97620" "I97638" "I97631" "I97621" "I9742" "I97418" "I97411" "I97611" "I97410" "I97610" "I97630" "I97410
Cardiac tamponade	Any cardiac tamponade after PFO occluder device placement	"I314"
